# Antibiotic‐loaded bone cement is associated with a reduction of the risk of revision of total knee arthroplasty: Analysis of the Catalan Arthroplasty Register

**DOI:** 10.1002/ksa.12361

**Published:** 2024-07-21

**Authors:** Sergi Gil‐Gonzalez, Borja Velasco‐Regúlez, Jesus Cerquides, Pedro Hinarejos, Joan Carles Monllau, Xavier Pelfort

**Affiliations:** ^1^ Institut d'investigació i Innovació Parc Taulí (I3PT‐CERCA), Parc Taulí Hospital Universitari Universitat Autònoma de Barcelona Sabadell Spain; ^2^ Data and Artificial Intelligence Agency for Health Quality and Assessment of Catalonia (AQuAS) Barcelona Spain; ^3^ Learning systems Artificial Intelligence Research Institute (IIIA‐CSIC) Bellaterra Spain; ^4^ Department of Orthopaedic Surgery, Parc de Salut Mar, Hospital de la Esperanza Universitat Autònoma de Barcelona Barcelona Spain

**Keywords:** antibiotic‐loaded bone cement, peri‐prosthetic joint infection, plain cement, total knee arthroplasty

## Abstract

**Purpose:**

The purpose of this study was to analyse the impact on peri‐prosthetic joint infection (PJI) rate and prosthetic survival using antibiotic‐loaded bone cement (ALBC) versus plain cement during total knee arthroplasty (TKA).

**Methods:**

A retrospective cohort study was conducted. The main data source was the Catalan Arthroplasty Register (RACat). TKAs with surgery date between 1 January 2011 and 31 December 2020 were analysed and followed up until 31 December 2023. The main variable of interest was the type of cement (ALBC vs. plain cement), and several endpoints (septic revision, aseptic revision, and all‐cause revision) were considered. The analysed outcomes were revision rates, survival rates and risk factors' hazard ratios (HR).

**Results:**

A total of 22,781 TKAs were analysed, 13,125 (57.6%) of them with plain cement and 9656 (42.4%) with ALBC. The septic revision rate was lower in the ALBC group after 3 months of follow‐up (0.52% vs. 0.78%, *p* value = 0.04). Prosthetic survival with respect to the aseptic revision endpoint was also higher for the ALBC group during the whole follow‐up period (~158 months). Regarding risk factors for infection, ALBC showed a protective effect, HR: 0.53 (0.44, 0.63), while sex (being male) and the analysed comorbidities increased the risk.

**Conclusions:**

ALBC is associated with a reduction in both the septic revision and the aseptic revision rate after TKA, and thus with higher prosthetic survival.

**Level of Evidence:**

Level III, Therapeutic, retrospective.

**Abbreviations**
ALBC: antibiotic‐loaded bone cement
BMSD: basic minimum set of data
CatSalut: Catalan health service
CPHM: Cox's proportional hazards model
ICS: Catalan institute of health
PJI: peri‐prosthetic joint infection
RACat: Catalan arthroplasty register
TKA: total knee arthroplasty
VINCat: Catalan nosocomial infection surveillance programme

## INTRODUCTION

Peri‐prosthetic joint infection (PJI) is a major complication of total knee arthroplasty (TKA), which happens between 1% and 2% of the cases [10]. Patients who are affected by this complication usually need revision surgery, which has a big negative impact on the quality of life and leads to decreased patient satisfaction [11]. The last decades have witnessed many contributions and improvements aimed at reducing the rate of PJI. Some examples are the use of prophylactic antibiotics, improvements in orthopaedic theatres and modifications in preoperative patient preparation [28]. In the case of total hip arthroplasty, the usage of antibiotic‐loaded bone cement (ALBC) during primary arthroplasty has also been shown to decrease the rate of PJI [9, 14]. However, in the case of TKA, the evidence of the benefit of that strategy is inconclusive [21, 22]. Downsides such as the possibility of altering the mechanical properties of the cement, the generation of antibiotic microbial resistance or the increasing cost [7, 15, 18] cause a lack of consensus about this intervention across countries, and there is substantial variability in the findings reported by studies carried out in different countries or regions [21]. Places like the United Kingdom or the Scandinavian countries use ALBC in primary TKA in more than 90% of cases, but this percentage is much lower in places like the United States, Spain or Russia [30]. Furthermore, patients' preoperative characteristics can also be associated with the risk of developing PJI. Gender, age, previous surgeries and comorbidities, such as diabetes, obesity or inflammatory diseases could increase the probability of septic complications [26, 35].

The objective of this study was to analyse the data of the Catalan arthroplasty registry, the largest analysed registry of southern Europe, with the hypothesis that ALBC would not be associated with reductions in septic nor aseptic revision rates. It was also a secondary goal of the study to assess the impact of other risk factors, such as the patient's gender, age, comorbidities, and so forth.

## METHODS

A retrospective cohort study was carried out, using data from the Catalan Arthroplasty Register (RACat), the Catalan nosocomial infection surveillance programme (VINCat), the Basic Minimum Set of Data (BMSD) of hospital discharges and databases of primary care. All data sources were managed by the Catalan health service (CatSalut) and the Catalan Institute of Health (ICS). The main source of data was RACat [2]. This regional, population‐based registry gathers information about knee and hip arthroplasties. It started its activity in 2005, and was a voluntary registry until 2015, when it became mandatory. It contains information from 51 out of the 56 public hospitals of Catalonia that perform TKAs under the coverage of CatSalut. VINCat contains information about hospital care‐related infections, is a voluntary registry and receives information from all the major hospitals of Catalonia. The BMSD of hospital discharges and the databases of primary care contain information about hospital activity and primary care assistance, such as diagnoses and procedures. They are mandatory registries, and thus they are considered the *golden standard* of the database.

**Figure 1:**
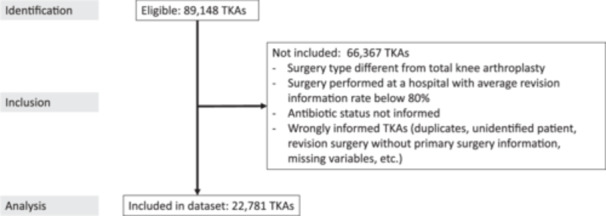
STROBE diagram of the study. The starting point consisted of 89,148 eligible TKAs. Finally 22,781 TKAs were included in the data set. TKA, total knee arthroplasty.

The starting point was the information on all knee arthroplasties contained in RACat with surgery dates between 1 January 2011 and 31 December 2020. The follow‐up was until 31 December 2023. Partially cemented arthroplasties and uni‐compartment arthroplasties were excluded, keeping only fully cemented TKAs. In addition, TKAs coming from hospitals with a low percentage of reported procedures were excluded, to avoid introducing biases. To compute the percentage of reporting for each hospital, first, we linked the RACat and the BMSD, requiring an exact match for patient identifier, surgery type and date of surgery. Then, the percentage of reported procedures was computed as the fraction of procedures registered by RACat with respect to those registered by BMSD. The threshold was set at 80% (see the Supporting Information for a justification of this value). Finally, TKAs with wrong or incomplete information were filtered out. This process resulted in 22,781 TKAs for final analysis. Figure 1 shows the STROBE [8] flow diagram of this process.

The main variable of interest was the use of ALBC or plain cement during primary TKA. The main outcomes of interest were prosthetic survival and revision rate, and the considered event was the revision, that is, a surgery after the primary surgery where at least one component was revised (excluding the patellar component). Depending on the diagnostic of the revision, three endpoints were specified: septic revision (revision due to infection), aseptic revision (revision due to a cause other than infection) and all‐cause revision. In the remainder of this text, we may use the terms *septic revision* or simply *infection* interchangeably. The preoperative characteristics, covariates or risk factors considered were patient's age, as a continuous variable in years; sex assigned at birth, as a dichotomous variable (woman or man); obesity, diabetes, rheumatoid arthritis and alcohol abuse, as dichotomous variables (yes or no); smoking status, as a categorical variable (smoker, nonsmoker, former smoker; missing values treated as nonsmoker); body mass index (BMI) as a continuous variable in kg/m2 (missing values imputed with the average stratified by age group); Charlson comorbidity index and Elixhauser index, as continuous variables; hospital category, as a categorical ordinal variable with five categories (between 1 and 5); primary surgery year, as a categorical ordinal variable (from 2011 to 2020); surgery duration, as a continuous variable in minutes (missing values assigned with the average stratified by the hospital. Note that the hospital category classifies hospitals regarding their size and specialization level, and it is a categorization established by CatSalut. Category 1 is for high‐technology, reference hospitals, while Category 5 is for regional, basic hospitals. Alcohol abuse was defined as in the definition of the Elixhauser index [23].

We employed estimators and models that are widely used in survival analysis, such as the Kaplan−Meier estimator and the Cox proportional hazards models (see the Supporting Information for a justification of this choice). For the nonparametric models, estimators were computed for the two groups defined by the variable of interest, that is, ALBC or plain cement. *Z*‐tests, *t* tests and Log‐rank tests were used for determining whether observed differences between groups were statistically significant, with confidence value *⍺* = 0.05.

The data were extracted, linked and analysed within the Data Analysis Program for Research and Innovation in Healthcare (PADRIS), managed by AQuAS, the Agency for Health Quality and Assessment of Catalonia.

National ethics committee approval with number PR186/19 and Advisory Committee of RACat approval were obtained for this study.

## RESULTS

Table 1 presents a descriptive analysis of the most relevant preoperative characteristics of the study population, stratified by the variable of interest (plain cement/ALBC). Overall, plain cement was used in 9656 (42.4%) cases and ALBC in 13,125 (57.6%) cases. In the ALBC group, gentamicin was used in 12703 (96.78%) cases, tobramycin in 410 (3.12%) cases and erythromycin in 12 (0.09%) cases. Small but statistically significant differences were found for sex and antibiotic usage (among females, 42.97% received plain cement and 57.03% ALBC, while among males, the percentages were 41.02% and 58.98%, respectively), and for age and antibiotic usage. No significant differences were found regarding the analysed comorbidities individually and antibiotic usage, although the small differences observed in the Charlson and Elixhauser indexes were statistically significant. Finally, larger differences were found for the variables of smoking status, surgery year and hospital category. The most significant one is the steady increase in ALBC usage over the years, going from 46.74% in 2011 to 84.16% in 2020 (of all surgeries in each year, respectively).

**Table 1:** Preoperative characteristics of the study population, stratified by the cement type.

Characteristic	Categories	Plain cement	ALBC	*p* Value
		*n* (%)		
Sex	Female	6843.0 (42.97%)	9081.0 (57.03%)	<0.01
	Male	2813.0 (41.02%)	4044.0 (58.98%)	
		mean (SD)		
Age (years)		72.24 (7.61)	71.94 (8.01)	<0.01
Hospital category	1	2246.0 (49.50%)	2291.0 (50.50%)	<0.01
	2	175.0 (4.20%)	3994.0 (95.80%)	
	3	3232.0 (48.56%)	3423.0 (51.44%)	
	4	3995.0 (56.32%)	3099.0 (43.68%)	
	5	8.0 (2.45%)	318.0 (97.55%)	
		mean (SD)		
Surgery duration		89.82 (14.40)	89.98 (21.21)	n.s.
Charlson index		0.41 (0.76)	0.46 (0.79)	<0.01
Elixhauser index		1.34 (1.18)	1.41 (1.20)	<0.01
		*n* (%)		
Obesity	No	8509.0 (42.49%)	11,518.0 (57.51%)	n.s.
	Yes	1147.0 (41.65%)	1607.0 (58.35%)	
Diabetes	No	8112.0 (42.68%)	10,893.0 (57.32%)	0.04
	Yes	1544.0 (40.89%)	2232.0 (59.11%)	
Rheumatoid arthritis	No	9460.0 (42.47%)	12,817.0 (57.53%)	n.s.
	Yes	196.0 (38.89%)	308.0 (61.11%)	
Alcohol abuse	No	9601.0 (42.43%)	13,025.0 (57.57%)	n.s.
	Yes	55.0 (35.48%)	100.0 (64.52%)	
Smoking status	Nonsmoker	7985.0 (43.37%)	10,426.0 (56.63%)	<0.01
	Smoker	555.0 (39.67%)	844.0 (60.33%)	
	Former smoker	1116.0 (37.56%)	1855.0 (62.44%)	
		mean (SD)		
Body mass index		31.82 (4.57)	31.76 (4.89)	n.s.

Abbreviation: ALBC, antibiotic‐loaded bone cement.

**Figure 2:**
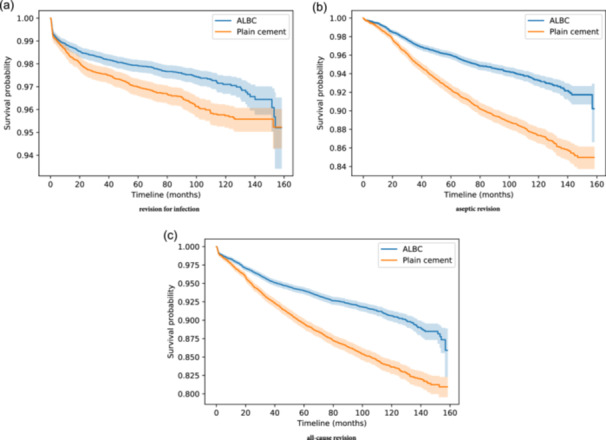
Kaplan−Meier curves for three endpoints: (a) revision for infection, (b) aseptic revision and (c) all‐cause revision. Significant differences in survival rates are observed in all three cases, in favour of the ALBC group. ALBC, antibiotic‐loaded bone cement.

The follow‐up period was 158 months (approximately 13 years). Figure 2 shows the Kaplan−Meier survival curves for three endpoints, septic revision (a), aseptic revision (b) and all‐cause revision (c), for the whole duration of the follow‐up period. The ALBC group showed higher survival values across study time for all endpoints (*p* value < 0.05 in all cases). The survival values (with 95% confidence intervals in brackets) at the end of the follow‐up period were 95.2% (93.4%, 96.5%) for ALBC and 95.2% (94.3%, 96%) for plain cement, in the case of septic revision; 90.2% (86.7%, 92.9%) for ALBC and 85% (83.8%, 86.1%) for plain cement, in the case of aseptic revision; and 85.9% (82.3%, 88.8%) for ALBC and 81% (79.6%, 82.2%) for plain cement, in the case of all‐cause revision.

**Figure 3:**
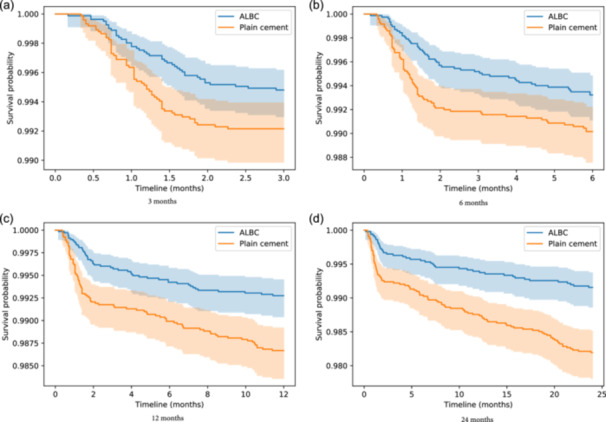
Kaplan−Meier survival curves for infection, for different follow‐up times: (a) 3 months, (b) 6 months, (c) 12 months and (d) 24 months. Differences are observed for all four scenarios, which become larger with the follow‐up time. ALBC, antibiotic‐loaded bone cement.

Table 2 and Figure 3 show the results of infection rates and Kaplan−Meier survival curves for both ALBC and plain cement groups, for the endpoint of septic revision and shorter follow‐up times (3, 6, 12 and 24 months, respectively). Uncensored data was used for this analysis, reducing the risk of bias of the Kaplan−Meier estimator. The infection rate was lower for the ALBC group in all cases with statistically significant differences (*p* value < 0.05 in all cases).

**Table 2:** Infection rates for short follow‐up times, stratified by the cement type.

	Cement type		
Follow‐up time	Plain cement %	ALBC %	*p* Value
3 months	0.78	0.52	0.04
6 months	0.98	0.68	0.04
12 months	1.33	0.72	<0.01
24 months	1.79	0.84	<0.01

Abbreviation: ALBC, antibiotic‐loaded bone cement.

**Figure 4:**
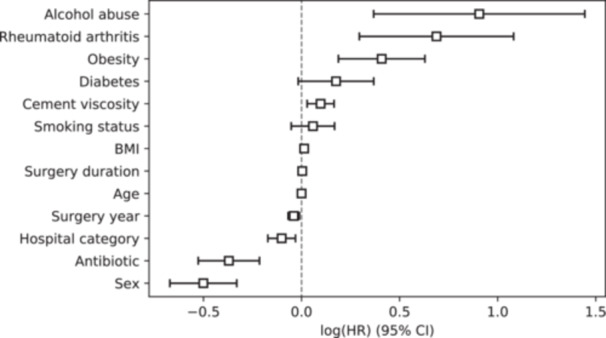
Boxplot of the hazard ratios of the Cox proportional hazards model with preoperative covariates in decreasing order.

Figure 4 shows a boxplot of the hazard ratios (HR) of the different covariates and the variable of interest (ALBC/plain cement) for septic revision, obtained by fitting Cox's proportional hazards model. The associated numerical information can be found in Table 3. ALBC was associated with a protective effect over infection (HR: 0.53 (0.44, 0.63), *p* value < 0.01). Other covariates, such as alcohol abuse, rheumatoid arthritis, obesity, diabetes and surgery year, were associated with a higher rate of infection (although Diabetes was not statistically significant by a small margin). Hospital category and sex showed a protective effect. Smoking status, BMI, surgery duration and age showed small or statistically not significant effects. Risk factor analysis for the endpoints of aseptic revision and all‐cause revision can be found in the Supporting Information.

**Table 3:** Cox proportional hazards model's results for endpoint infection.

			Event count	No event count	
		Hazard ratio	658	22,123	*p* Value
Antibiotic	Plain cement	ref	344	9312	<0.01
	ALBC	0.53 (0.44, 0.63)	314	12,811	
Sex	Male	Ref	270	6587	<0.01
	Female	0.61 (0.52, 0.73)	388	15,536	
Age		1.00 (0.99, 1.01)	71.54 (8.32)	72.08 (7.83)	n.s.
Hospital category	1	1.11 (1.19,1.03)	129	4408	0.01
	2	Ref	140	4029	
	3	1.22 (1.41,1.06)	208	6447	
	4	0.90 (0.84,0.97)	173	6921	
	5	0.82 (0.71,0.94)	8	318	
Surgery duration		1.00 (1.00, 1.01)	92.71 (31.74)	89.83 (18.09)	<0.01
Alcohol abuse	No	ref	644	21,982	<0.01
	Yes	2.31 (1.35, 3.96)	14	141	
Diabetes	No	Ref	525	18,480	<0.01
	Yes	1.25 (1.03, 1.51)	133	3643	
Obesity	No	ref	541	19,486	<0.01
	Yes	1.61 (1.29, 2.00)	117	2637	
Rheumatoid arthritis	No	Ref	632	21,645	<0.01
	Yes	1.92 (1.29, 2.84)	26	478	
Smoking status	Nonsmoker	ref	499	17,912	ref
	Smoker	1.13 (0.83, 1.54)	51	1348	n.s.
	Former smoker	1.10 (0.88, 1.38)	108	2863	n.s.
BMI		1.01 (0.97, 1.03)	32.26 (5.16)	31.77 (4.74)	n.s.

Abbreviations: ALBC, antibiotic‐loaded bone cement; BMI, body mass index.

## DISCUSSION

The most important finding of this study was that ALBC was associated with lower septic and aseptic revision rates after TKA, and thus with higher prosthetic survival, in the largest analysed TKA registry of southern Europe to date. The suggested mechanism for the protective effect of ALBC against infection is that the initial concentration of antibiotic released when performing the TKA would be enough to prevent bacterial biofilm formation [3, 14, 17].

The evidence of the benefits of using ALBC during TKA is inconclusive in the scientific literature, with some studies reaching conclusions in favour of it [16, 17], others against [27, 33] and others finding no differences [5, 13].

Our results align with those of the study with the largest sample size coming from a single database, the National Joint Registry from England and Wales [16]. In that work, the vast majority of analysed TKAs (93%) belonged to the ALBC group, and that circumstance also happened in other studies whose results are aligned with ours [17]. In the present work, nevertheless, the distribution between groups was more balanced: 57.6% in the ALBC group and 42.4% in the plain cement group.

Among the studies that found negative or no effect of ALBC, some performed the analysis in a sequential way [33], first doing a univariate analysis and then doing a multivariate analysis with factors that had achieved statistical significance in the first step. On the contrary, our analysis was carried out in a multivariate manner with all the variables in a single step. Some works may have suffered from confounding bias [28], as patients in the ALBC group had significantly higher values of risk factors, such as Diabetes Mellitus or ASA grade. This was not the case in our registry. Finally, some other studies used antibiotics, such as erythromycin and colistin [13], which are less routinely used than gentamicin, the predominant antibiotic in our database.

The meta‐analyses that have been published on the topic to date have shown, in general, no statistically significant differences in infection rates between groups [18, 22, 23]. Nevertheless, in the largest and most recent multiregistry meta‐analysis found in the literature [23], four out of nine registries showed results aligned with ours' (i.e., in favour of ALBC), and two of them achieved statistical significance. It is worth mentioning that such meta‐analysis considered the underrepresentation of some regions of the world a potential limitation for the generalizability of its findings, and yet the southern European region was rather underrepresented (both in that study and in the literature in general). In another meta‐analysis [22] no statistically significant differences between groups were found, although it is interesting to note that the two largest studies included did report them, both in favour of ALBC. Most of the largest studies analysed in that meta‐analysis have already been discussed in this section.

A secondary finding in our study was that the ALBC group also showed lower aseptic revision rates and higher prosthetic survival than the plain cement group. These results were aligned with the findings of several works in the literature [4, 5, 16], with the plausible explanation that ALBC could have acted as a protective factor against subclinical infections misclassified as aseptic loosening. Other studies found no effect or a negative one, suggesting that it could be caused by the worsening of the mechanical properties of the prostheses due to the ALBC. The evidence for that mechanism was found mostly in vitro [20, 25], and we did not observe it in our data. Some other works indicate that wrongly classified subclinical infections could bias the results of aseptic revision rates [6, 24].

Previous works have also analysed the associated risk of preoperative characteristics with prosthetic survival [17, 29, 31, 35]. In our data, sex (being male) was found to be a risk factor for infection after TKA, in line with other works [13, 19, 26]. Alcohol abuse, diabetes, obesity and rheumatoid arthritis were also risk factors for infection. Regarding obesity, a study indicated that local adiposity could be a better predictor for infection than the BMI [12], but our registry did not contain that information. Being a smoker or a former smoker was also associated with a higher risk of infection, although this variable did not achieve statistical significance. The results on all these risk factors were in line with those of the largest meta‐analysis carried out to date [32], except for the fact that we did not find age to be a protective factor.

Our study has limitations. First, RACat data completeness can vary for each hospital. We tried to overcome this by analysing TKAs performed in hospitals that had at least an 80% of prosthetic revision reporting rate, but the limitation was still present. Second, to identify PJI, septic revision diagnostic categories of RACat were used. Infections that were not treated surgically, whether superficial or deep, were not identified by this method. Nevertheless, this limitation is shared with other studies, making results comparable in principle. Finally, inputted values for the missing values of some of the available confounders, or the effect of other, unexplored confounders (such as surgical time, individual surgeons, individual hospitals or others) could have introduced bias in the results.

Overall, the combination of our results in favour of ALBC with some others indicating that it is cost‐effective [1] and that its use is not associated with bacterial resistance [34] brings evidence in favour of its routine use. We are aware that the most recent meta‐analyses do not find significant benefits, and we consider that larger pragmatic trials are required.

## CONCLUSIONS

In our study, the use of ALBC was associated with a lower rate of septic and aseptic revision, and thus with higher prosthetic survival, suggesting that its routine use was beneficial. In addition, the main risk factors for PJI were sex (being male), obesity, diabetes, rheumatoid arthritis and alcohol abuse.

## AUTHOR CONTRIBUTIONS

**Sergi Gil‐Gonzalez**: Conceptualization; methodology; formal analysis and investigation; writing—review and editing. **Borja Velasco‐Regúlez**: Methodology; formal analysis and investigation; writing—original draft preparation. **Jesus Cerquides**: Methodology; formal analysis and investigation; writing—review and editing; funding acquisition; supervision. **Pedro Hinarejos**: Conceptualization; supervision. **Joan Carles Monllau**: Conceptualization; supervision. **Xavier Pelfort**: Conceptualization; supervision.

## CONFLICT OF INTEREST STATEMENT

The authors declare no conflict of interest.

## ETHICS STATEMENT

National ethics committee approval with number PR186/19 and institutional review board were obtained for this study. The related documentation can be made available upon request.

## Data Availability

The data employed for this study was individual patient pseudo‐anonymized data, which was obtained under the coverage of the Data Analysis Program for Research and Innovation in Healthcare (PADRIS), managed by AQuAS, the Agency for Health Quality and Assessment of Catalonia. This data cannot be made available publicly.

## References

[1] Abdel Khalik, H. , Wood, T.J. , Tushinski, D.M. , Gazendam, A. , Petruccelli, D.T. , Bali, K. et al. (2023) Routine use of antibiotic‐laden bone cement in total knee arthroplasty is a cost‐effective practice in the single‐payer healthcare system. Knee Surgery, Sports Traumatology, Arthroscopy, 31(9), 3847–3853. Available from: 10.1007/s00167-023-07364-5 36905414

[2] Arias‐de la Torre, J. , Capdevila, A. , Martínez, O. , Domingo, L. , Marinelli, M. , Robles, N. et al. (2017) A decade of the Catalonian Arthroplasty Register (RACat): variability, exhaustivity, and survival of prostheses between 2005 and 2014. Revista Española de Cirugía Ortopédica y Traumatología (English Edition), 61(2), 70–81. Available from: 10.1016/j.recote.2017.02.005 28223094

[3] van de Belt, H.D. , Neut, D. , Schenk, W. , Horn, J.R. , Mei, H.C.D. & Busscher, H.J. (2000) Gentamicin release from polymethylmethacrylate bone cements and Staphylococcus aureus biofilm formation. Acta Orthopaedica Scandinavica, 71(6), 625–629Available from: 10.1080/000164700317362280 11145392

[4] Bendich, I. , Zhang, N. , Barry, J.J. , Ward, D.T. , Whooley, M.A. & Kuo, A.C. (2020) Antibiotic‐laden bone cement use and revision risk after primary total knee arthroplasty in US veterans. Journal of Bone and Joint Surgery, 102(22), 1939–1947. Available from: 10.2106/JBJS.20.00102 32890041

[5] Bohm, E. , Zhu, N. , Gu, J. , de Guia, N. , Linton, C. , Anderson, T. et al. (2014) Does adding antibiotics to cement reduce the need for early revision in total knee arthroplasty? Clinical Orthopaedics & Related Research, 472(1), 162–168. Available from: 10.1007/s11999-013-3186-1 23884803 PMC3889417

[6] Bozzo, A. , Ekhtiari, S. , Madden, K. , Bhandari, M. , Ghert, M. & Khanna, V. et al. (2022) Incidence and predictors of prosthetic joint infection following primary total knee arthroplasty: a 15‐year population‐based cohort study. The Journal of Arthroplasty, 37(2), 367–372. Available from: 10.1016/j.arth.2021.10.006 34678445

[7] Dunne, N. , Hill, J. , McAfee, P. , Todd, K. , Kirkpatrick, R. , Tunney, M. et al. (2007) In vitro study of the efficacy of acrylic bone cement loaded with supplementary amounts of gentamicin: effect on mechanical properties, antibiotic release, and biofilm formation. Acta Orthopaedica, 78(6), 774–785. Available from: 10.1080/17453670710014545 18236183

[8] von Elm, E. , Altman, D.G. , Egger, M. , Pocock, S.J. , Gøtzsche, P.C. & Vandenbroucke, J.P. (2008) The Strengthening the Reporting of Observational Studies in Epidemiology (STROBE) statement: guidelines for reporting observational studies. Journal of Clinical Epidemiology, 61(4), 344–349. Available from: 10.1016/j.jclinepi.2007.11.008 18313558

[9] Engesæter, L.B. , Espehaug, B. , Lie, S.A. , Furnes, O. & Havelin, L.I. (2006) Does cement increase the risk of infection in primary total hip arthroplasty? Revision rates in 56,275 cemented and uncemented primary THAs followed for 0‐16 years in the Norwegian Arthroplasty Register. Acta Orthopaedica, 77(3), 351–358. Available from: 10.1080/17453670610046253 16819671

[10] Frank, R. , Cross, M. & Della Valle, C. (2015) Periprosthetic joint infection: modern aspects of prevention, diagnosis, and treatment. Journal of Knee Surgery, 28(2), 105–112. Available from: 10.1055/s-0034-1396015 25409493

[11] Garvin, K.L. & Konigsberg, B.S. (2011) Infection following total knee arthroplasty: prevention and management. The Journal of Bone & Joint Surgery, 93(12), 1167–1175. Available from: 10.2106/00004623-201106150-00012 21776555

[12] Heifner, J.J. , Sakalian, P.A. , Rowland, R.J. & Corces, A. (2023) Local adiposity may be a more reliable predictor for infection than body mass index following total knee arthroplasty: a systematic review. Journal of Experimental Orthopaedics, 10(1), 110. Available from: 10.1186/s40634-023-00680-2 37930482 PMC10628095

[13] Hinarejos, P. , Guirro, P. , Leal, J. , Montserrat, F. , Pelfort, X. , Sorli, M.L. et al. (2013) The use of erythromycin and colistin‐loaded cement in total knee arthroplasty does not reduce the incidence of infection: a prospective randomized study in 3000 knees. Journal of Bone and Joint Surgery, 95(9), 769–774. Available from: 10.2106/JBJS.L.00901 23636182

[14] Hinarejos, P. , Guirro, P. , Puig‐Verdie, L. , Torres‐Claramunt, R. , Leal‐Blanquet, J. & Sanchez‐Soler, J. et al. (2015) Use of antibiotic‐loaded cement in total knee arthroplasty. World Journal of Orthopedics, 6(11), 877–885. Available from: 10.5312/wjo.v6.i11.877 26716084 PMC4686435

[15] Hoskins, T. , Shah, J.K. , Patel, J. , Mazzei, C. , Goyette, D. , Poletick, E. et al. (2020) The cost‐effectiveness of antibiotic‐loaded bone cement versus plain bone cement following total and partial knee and hip arthroplasty. Journal of Orthopaedics, 20, 217–220. Available from: 10.1016/j.jor.2020.01.029 32051672 PMC7005330

[16] Jameson, S.S. , Asaad, A. , Diament, M. , Kasim, A. , Bigirumurame, T. , Baker, P. et al. (2019) Antibiotic‐loaded bone cement is associated with a lower risk of revision following primary cemented total knee arthroplasty: an analysis of 731,214 cases using National Joint Registry data. The Bone & Joint Journal, 101–B, 1331–1347. Available from: 10.1302/0301-620X.101B11.BJJ-2019-0196.R1 31674244

[17] Jämsen, E. , Huhtala, H. , Puolakka, T. & Moilanen, T. (2009) Risk factors for infection after knee arthroplasty. A register‐based analysis of 43,149 cases. The Journal of Bone and Joint Surgery‐American Volume, 91(1), 38–47. Available from: 10.2106/JBJS.G.01686 19122077

[18] King, J.D. , Hamilton, D.H. , Jacobs, C.A. & Duncan, S.T. (2018) The hidden cost of commercial antibiotic‐loaded bone cement: a systematic review of clinical results and cost implications following total knee arthroplasty. The Journal of Arthroplasty, 33(12), 3789–3792. Available from: 10.1016/j.arth.2018.08.009 30217400

[19] Kurtz, S.M. , Ong, K.L. , Lau, E. , Bozic, K.J. , Berry, D. & Parvizi, J. (2010) Prosthetic joint infection risk after TKA in the Medicare population. Clinical Orthopaedics & Related Research, 468(1), 52–56. Available from: 10.1007/s11999-009-1013-5 19669386 PMC2795807

[20] Lautenschlager, E.P. , Jacobs, J.J. , Marshall, G.W. & Meyer, P.R. (1976) Mechanical properties of bone cements containing large doses of antibiotic powders. Journal of Biomedical Materials Research, 10, 929–938. Available from: 10.1002/jbm.820100610 993228

[21] Leta, T.H. , Lie, S.A. , Fenstad, A.M. , Lygre, S.H.L. , Lindberg‐Larsen, M. , Pedersen, A.B. et al. (2024) Periprosthetic Joint Infection After Total Knee Arthroplasty With or Without Antibiotic Bone Cement, JAMA Netw Open, 7(5), e2412898. 10.1001/jamanetworkopen.2024.12898 38780939 PMC11117087

[22] Li, H.Q. , Li, P.C. , Wei, X.C. & Shi, J.J. (2022) Effectiveness of antibiotics loaded bone cement in primary total knee arthroplasty: a systematic review and meta‐analysis. Orthopaedics & Traumatology: Surgery & Research, 108(5), 103295. Available from: 10.1016/j.otsr.2022.103295 35552043

[23] Lix, L. , Smith, M. , Pitz, M. , Ahmed, R. , Quon, H. , Griffith, J. et al. (2016) Cancer data linkage in Manitoba: expanding the infrastructure for research. Winnipeg, MB: Manitoba Centre for Health Policy.

[24] Maathuis, P.G.M. , Neut, D. , Busscher, H.J. , van der Mei, H.C. & van Horn, J.R. (2005) Perioperative contamination in primary total hip arthroplasty. Clinical Orthopaedics and Related Research, 433, 136–139. Available from: 10.1097/01.blo.0000149997.14631.0c 15809639

[25] Moran, J.M. , Greenwald, A.S. & Matejczyk, M.B. (1979) Effect of gentamicin on shear and interface strengths of bone cement. Clinical Orthopaedics and Related Research, 141, 96–101. 477129

[26] Namba, R.S. , Inacio, M.C.S. & Paxton, E.W. (2013) Risk factors associated with deep surgical site infections after primary total knee arthroplasty: an analysis of 56,216 knees. Journal of Bone and Joint Surgery, 95(9), 775–782. Available from: 10.2106/JBJS.L.00211 23636183

[27] Namba, R.S. , Chen, Y. , Paxton, E.W. , Slipchenko, T. & Fithian, D.C. (2009) Outcomes of routine use of antibiotic‐loaded cement in primary total knee arthroplasty. The Journal of Arthroplasty, 24(6 Suppl), 44–47. Available from: 10.1016/j.arth.2009.05.007 19577881

[28] Parvizi, J. , Cavanaugh, P.K. & Diaz‐Ledezma, C. (2013) Periprosthetic knee infection: ten strategies that work. Knee Surgery & Related Research, 25(4), 155–164. Available from: 10.5792/ksrr.2013.25.4.155 24368992 PMC3867607

[29] Rand, J.A. , Trousdale, R.T. , Ilstrup, D.M. & Harmsen, W.S. (2003) Factors affecting the durability of primary total knee prostheses. The Journal of Bone and Joint Surgery‐American Volume, 85(2), 259–265. Available from: 10.2106/00004623-200302000-00012 12571303

[30] Randelli, P. , Evola, F.R. , Cabitza, P. , Polli, L. , Denti, M. & Vaienti, L. (2010) Prophylactic use of antibiotic‐loaded bone cement in primary total knee replacement. Knee Surgery, Sports Traumatology, Arthroscopy, 18(2), 181–186. Available from: 10.1007/s00167-009-0921-y 19795106

[31] Rassir, R. , Sierevelt, I.N. , van Steenbergen, L.N. & Nolte, P.A. (2020) Is obesity associated with short‐term revision after total knee arthroplasty? An analysis of 121,819 primary procedures from the Dutch Arthroplasty Register. The Knee, 27(6), 1899–1906. Available from: 10.1016/j.knee.2020.09.020 33220579

[32] Resende, V.A.C. , Neto, A.C. , Nunes, C. , Andrade, R. , Espregueira‐Mendes, J. & Lopes, S. (2021) Higher age, female gender, osteoarthritis and blood transfusion protect against periprosthetic joint infection in total hip or knee arthroplasties: a systematic review and meta‐analysis. Knee Surgery, Sports Traumatology, Arthroscopy, 29(1), 8–43. Available from: 10.1007/s00167-018-5231-9 30413860

[33] Tayton, E.R. , Frampton, C. , Hooper, G.J. & Young, S.W. (2016) The impact of patient and surgical factors on the rate of infection after primary total knee arthroplasty: an analysis of 64,566 joints from the New Zealand Joint Registry. The Bone & Joint Journal, 98–B, 334–340. Available from: 10.1302/0301-620X.98B3.36775 26920958

[34] Tootsi, K. , Heesen, V. , Lohrengel, M. , Enz, A.E. , Illiger, S. , Mittelmeier, W. et al. (2022) The use of antibiotic‐loaded bone cement does not increase antibiotic resistance after primary total joint arthroplasty. Knee Surgery, Sports Traumatology, Arthroscopy, 30(9), 3208–3214. Available from: 10.1007/s00167-021-06649-x PMC9418265 34244827

[35] Wall, C.J. , Vertullo, C.J. , Kondalsamy‐Chennakesavan, S. , Lorimer, M.F. & de Steiger, R.N. (2022) A prospective, longitudinal study of the influence of obesity on total knee arthroplasty revision rate: results from the Australian Orthopaedic Association National Joint Replacement Registry. The Journal of Bone and Joint Surgery‐American Volume, 104(15), 1386–1392. Available from: http://10.2106/JBJS.21.01491 35703139 10.2106/JBJS.21.01491

